# Joint effect of longevity-associated mitochondrial DNA 5178 C/A polymorphism and alcohol consumption on risk of hyper-LDL cholesterolemia in middle-aged Japanese men

**DOI:** 10.1186/1476-511X-10-105

**Published:** 2011-06-25

**Authors:** Teruyoshi Kawamoto, Akatsuki Kokaze, Mamoru Ishikawa, Naomi Matsunaga, Kanae Karita, Masao Yoshida, Naoki Shimada, Tadahiro Ohtsu, Takako Shirasawa, Hirotaka Ochiai, Taku Ito, Hiromi Hoshino, Yutaka Takashima

**Affiliations:** 1 Department of Public Health, Showa University School of Medicine, 1-5-8 Hatanodai, Shinagawa-ku, Tokyo 142-8555, Japan; 2 Department of Public Health, Kyorin University School of Medicine, 6-20-2 Shinkawa, Mitaka-shi, Tokyo 181-8611, Japan; 3 Mito Red Cross Hospital, 3-12-48 Sannomaru, Mito-shi, Ibaraki 310-0011, Japan

## Abstract

**Background:**

Combined effects between mitochondrial DNA 5178 (Mt5178) C/A polymorphism and alcohol consumption on the risk of hypertension or hyperuricemia have been reported. The objective of this study was to investigate whether Mt5178 C/A polymorphism modulates the effects of alcohol consumption on the risk of dyslipidemia.

**Methods:**

A total of 394 male subjects were selected from among individuals visiting the hospital for regular medical check-ups. After Mt5178 C/A genotyping, a cross-sectional study assessing the combined effect of Mt5178 polymorphism and alcohol consumption on the risk of dyslipidemia was conducted.

**Results:**

For men with Mt5178C, alcohol consumption was significantly and negatively associated with the risk of hyper-low-density lipoprotein (LDL) cholesterolemia (serum LDL cholesterol ≥ 140 mg/dl) (*P *for trend = 0.015). After adjustment for age, body mass index (BMI), habitual smoking, coffee consumption and use of antihypertensive medicine, the odds ratio (OR) for hyper-LDL cholesterolemia was significantly lower in daily drinkers with Mt5178C than non-drinkers with Mt5178C (OR = 0.360, 95% confidence intervals: 0.153-0.847). A significant and negative association between alcohol consumption and serum LDL cholesterol levels was also observed in Mt5178C genotypic men (*P *for trend < 0.01). On the other hand, the association between Mt5178A genotype and risk of hyper-LDL cholesterolemia does not appear to depend on alcohol consumption.

**Conclusions:**

For Mt5178C genotypic men, alcohol consumption may reduce the risk of hyper-LDL cholesterolemia.

## Introduction

Mitochondrial DNA 5178 (Mt5178) C/A polymorphism, also known as NADH dehydrogenase subunit-2 237 (ND2-237) Leu/Met, is a longevity-associated mitochondrial DNA polymorphism [1-3]. The frequency of the Mt5178A genotype is significantly higher in Japanese centenarians than in the general population [1]. Japanese individuals with Mt5178A are more resistant to lifestyle-related adult-onset diseases, such as hypertension [4], diabetes [5], myocardial infarction [6,7] and cerebrovascular disorders [8], than those with Mt5178C. This polymorphism is also associated with serum lipid levels [9] and fasting plasma glucose levels [10].

A combined effect of Mt5178 C/A polymorphism and alcohol consumption on serum lipid levels has been reported [11]. Alcohol consumption is negatively associated with serum triglyceride levels in healthy male subjects with Mt5178A and with serum low-density lipoprotein (LDL) cholesterol levels in those with Mt5178C [11]. Previous studies have reported the joint effects of Mt5178 C/A polymorphism and alcohol intake on the risk of hypertension or hyperuricemia in middle-aged Japanese men. For men with the Mt5178C genotype, daily alcohol consumption may increase the risk of hypertension [4] or hyperuricemia [12]. However, there have been no reports on the association between Mt5178 C/A polymorphism and alcohol intake on the risk of dyslipidemia, which is clinically important for the prevention of atherosclerotic diseases [13].

The objective of this study was to investigate whether there is a joint effect of Mt5178 C/A polymorphism and alcohol consumption on the risk of dyslipidemia in middle-aged Japanese male subjects.

## Materials and methods

### Study subjects

Participants were recruited from among individuals visiting the Mito Red Cross Hospital for regular medical check-ups between August 1999 and August 2000. This study was conducted in accordance with the Declaration of Helsinki and was approved by the Ethics Committee of the Kyorin University School of Medicine. Written informed consent was obtained from 602 volunteers before participation. Because the number of women was insufficient for classification into groups based on Mt5178 C/A genotype and alcohol consumption habits, women were excluded. Patients taking antihyperlipidemic medication were excluded, and diabetic patients were also excluded because of their higher prevalence of dyslipidemia when compared to non-diabetic patients [14]. Thus, 406 men, including patients using antihypertensive medication, were enrolled in the study. Twelve individuals with unclear data were also excluded. Therefore, subjects comprised 394 Japanese men (age, 53.9 ± 7.9 years; mean ± SD).

### Clinical characteristics of subjects

Determination of blood chemical and physical data was conducted as described previously [9]. Serum total cholesterol levels, serum high-density lipoprotein (HDL) cholesterol levels, serum triglyceride levels, fasting plasma glucose levels and serum uric acid levels were measured using standard laboratory techniques. LDL cholesterol levels were calculated by Friedewald's formula [15]. Dyslipidemias were defined according to the Japan Atherosclerosis Society guidelines for the prevention of atherosclerotic cardiovascular diseases (hyper-LDL cholesterolemia was defined as serum LDL cholesterol ≥ 140 mg/dl, hypo-HDL cholesterolemia was defined as serum HDL cholesterol < 40 mg/dl and hypertriglyceridemia was defined as serum triglyceride ≥ 150 mg/dl) [13]. The values presented for both systolic blood pressure and diastolic blood pressure were the averages of two measurements obtained by physicians. Body mass index (BMI) was defined as the ratio of subject weight (kg) to the square of subject height (m). A survey of alcohol consumption, habitual smoking, coffee intake and use of antihypertensive medication was performed by means of questionnaire. Alcohol consumption was classified based on drinking frequency (daily drinkers; occasional drinkers, which include those who drink several times per week or per month; and non- or ex-drinkers). Habitual smoking was classified as non- or ex-smokers and current smokers. Coffee consumption was classified based on number of cups of coffee per day (less than one cup per day; one, two or three cups per day; and more than four cups per day). For use of antihypertensive medication, subjects were classified as taking no drug treatment or taking medicine.

### Genotyping

DNA was extracted from white blood cells using the DNA Extractor WB kit (Wako Pure Chemical Industries, Japan). The Mt5178 C/A polymorphism was detected by polymerase chain reaction (PCR) and digestion with *Alu*I restriction enzyme. Primers were: forward 5'-CTTAGCATACTCCTCAATTACCC-3'; and reverse 5'-GTGAATTCTTCGATAATGGCCCA-3'. PCR was performed with 50 ng of genomic DNA in buffer containing 0.2 μmol/l each primer, 1.25 mmol/l dNTPs, 1.5 mmol/l MgCl_2_, and 1 U of Taq DNA polymerase. After an initial denaturation at 94°C for 5 min, PCR was conducted through 40 cycles as follows: denaturation at 94°C for 30 s; annealing at 60°C for 60 s; and polymerase extension at 72°C for 90 s. After cycling, a final extension at 72°C for 10 min was performed. PCR products were digested with *Alu*I restriction enzyme (Nippon Gene, Japan) at 37°C overnight, and were electrophoresed on 1.5% agarose gels stained with ethidium bromide for visualization under ultraviolet light. The absence of an *Alu*I site was designated as Mt5178A (279-bp fragment), and the presence of this restriction site was designated as Mt5178C (175-bp and 104-bp fragments).

### Statistical analyses

Statistical analyses were performed using SAS statistical software, version 9.1, for Windows (SAS Institute, Inc., Cary, NC, 2002). Multiple logistic regression analysis was used to calculate the odds ratio (OR) of dyslipidemia. Differences in serum lipid levels between groups by alcohol consumption were evaluated using the least square means calculated from analysis of covariance. Differences with *P *values of less than 0.05 were considered to be statistically significant.

## Results

No significant differences in biophysical or biochemical characteristics, or in frequency of dyslipidemia were observed between the Mt5178C and Mt5178A genotypes (Table 1).

**Table 1:** Clinical characteristics of study subjects by Mt5178 C/A genotype

	Mt5178C	Mt5178A	*P *value
	**N = 239**	**N = 155**	
	
Age (y)	54.3 ± 7.8	53.2 ± 7.8	0.171
Body Mass Index (kg/m^2^)	23.3 ± 2.8	23.5 ± 2.6	0.461
Systolic blood pressure (mmHg)	125.8 ± 15.9	125.7 ± 14.1	0.940
Diastolic blood pressure (mmHg)	74.0 ± 10.7	73.8 ± 9.1	0.829
Total cholesterol (mg/dl)	203.5 ± 34.3	201.9 ± 31.8	0.672
LDL cholesterol (mg/dl)	121.6 ± 34.8	117.9 ± 30.5	0.281
HDL cholesterol (mg/dl)	54.6 ± 13.6	56.3 ± 16.2	0.269
Triglyceride (mg/dl)	136.7 ± 91.1	139.5 ± 90.8	0.766
Fasting plasma glucose (mg/dl)	97.2 ± 9.4	97.6 ± 9.7	0.672
Uric acid (mg/dl)	5.98 ± 1.21	5.92 ± 1.21	0.673
Alcohol consumption (daily/occasional/non- or ex-) (%)	46.4/35.2/18.4	47.7/38.7/13.6	0.426
Coffee consumption (< 1 cup per day/1-3 cups per day/≥ 4 cups per day) (%)	44.8/46.0/9.2	36.8/51.6/11.6	0.274
Current smokers (%)	41.4	40.7	0.878
Antihypertensive medication use (%)	18.8	12.9	0.122
Hyper-LDL cholesterolemia (%)	26.4	25.8	0.903
Hypo-HDL cholesterolemia (%)	11.7	12.3	0.871
Hypertriglyceridemia (%)	29.7	29.7	0.995

LDL; low-density lipoprotein, HDL; high-density lipoprotein. Age, body mass index, systolic blood pressure, diastolic blood pressure, serum total cholesterol levels, serum LDL cholesterol levels, serum HDL cholesterol levels, serum triglyceride levels, fasting plasma glucose levels and serum uric acid levels are given as means ± S.D. All *P *values depict significance of differences between Mt5178C and Mt5178A. For alcohol consumption, coffee consumption, current smokers, antihypertensive medication use, hyper-LDL cholesterolemia, hypo-HDL cholesterolemia and hypertriglyceridemia, *P *values were calculated by chi-squared test.

Significant and negative associations between alcohol consumption and the risk of hyper-LDL cholesterol were observed in Mt5178C genotypic men (*P *for trend = 0.015 and adjusted *P *for trend = 0.014, respectively). The OR for hyper-LDL cholesterolemia was significantly lower in daily drinkers with Mt5178C than in non-drinkers with Mt5178C (OR = 0.408, 95% confidence intervals (CI): 0.188-0.888, *P *= 0.024) (Table 2). After adjustment for age, body mass index, habitual smoking, coffee consumption and use of antihypertensive medication, a significant OR remained (OR = 0.360, 95% CI: 0.153-0.847, *P *= 0.019). On the other hand, the association between Mt5178A genotype and hyper-LDL cholesterolemia does not appear to depend on alcohol consumption. No significant joint effect of Mt5178 C/A polymorphism and alcohol consumption on risk of hypo-HDL cholesterolemia or hypertrygliceridemia was observed (data not shown).

**Table 2:** Odds ratios (ORs) and 95% confidence intervals (CIs) for hyper-LDL cholesterolemia by Mt5178 C/A genotype and alcohol consumption

	Frequency			
			
Genotype and alcohol consumption	Normal LDL cholesterol (LDL cholesterol < 140 mg/dl)	Hyper-LDL cholesterolemia (LDL cholesterol ≥ 140 mg/dl)	OR (95% CI)	Adjusted OR† (95% CI)
Mt5178C				
				
Non- or ex-drinkers (%)	28 (63.6)	16 (36.4)	1 (reference)	1 (reference)
Occasional drinkers (%)	58 (69.1)	26 (30.9)	0.784 (0.364-1.692)	0.694 (0.290-1.660)
Daily drinkers (%)	90 (81.1)	21 (18.9)	0.408 (0.188-0.888)*	0.360 (0.153-0.847)*
			*P *for trend = 0.015	*P *for trend = 0.014
				
Mt5178A				
				
Non- or ex-drinkers (%)	16 (76.2)	5 (23.8)	1 (reference)	1 (reference)
Occasional drinkers (%)	45 (75.0)	15 (25.0)	1.067 (0.334-3.409)	1.190 (0.318-4.447)
Daily drinkers (%)	54 (73.0)	20 (27.0)	1.185 (0.384-3.660)	1.232 (0.361-4.206)
			*P *for trend = 0.730	*P *for trend = 0.558

LDL; low-density lipoprotein. †OR adjusted for age, body mass index, habitual smoking, coffee consumption and antihypertensive medication use.

*P < 0.05.

Bonferroni correction for multiple comparisons revealed that serum LDL levels were significantly lower in daily drinkers with Mt5178C than in non- or ex-drinkers with Mt5178C (*P *= 0.011) (Table 3). After adjusting for age and BMI, serum LDL cholesterol levels were also significantly lower in daily drinkers with Mt5178C than in non- or ex-drinkers with Mt5178C (*P *= 0.007). Moreover, after adjusting for age, BMI, habitual smoking, coffee consumption and use of antihypertensive medicine, serum LDL cholesterol levels were significantly lower in occasional drinkers with Mt5178C or daily drinkers with Mt5178C than in non- or ex-drinkers with Mt5178C (*P *= 0.049 and *P *= 0.008, respectively). As crude and adjusted *P*-values for trends reached statistically significant levels (*P *for trend < 0.01), a significant and negative association between alcohol consumption and serum LDL cholesterol levels was confirmed in subjects with Mt5178C genotype. On the other hand, no noticeable relationship between alcohol intake and serum LDL cholesterol levels was observed in those with the Mt5178A genotype.

**Table 3:** Serum LDL cholesterol levels in habitual alcohol consumption groups by Mt5178 C/A genotype

	Alcohol consumption			*P *for trend
		
	Non- or ex-drinkers	Occasional drinkers	Daily drinkers	
Mt5178C	N = 44	N = 84	N = 111	
				
LDL cholesterol	135.0 ± 5.2	120.5 ± 3.7	117.1 ± 3.3*	0.007
LDL cholesterol †	135.8 ± 5.2	120.4 ± 3.8	116.9 ± 3.3**	0.005
LDL cholesterol ‡	135.6 ± 6.3	119.5 ± 4.9*	116.4 ± 4.1**	0.006
				
Mt5178A	N = 21	N = 60	N = 74	
				
LDL cholesterol	121.4 ± 6.7	118.5 ± 4.0	116.5 ± 3.6	0.506
LDL cholesterol †	122.1 ± 6.7	118.4 ± 3.9	116.3 ± 3.5	0.445
LDL cholesterol ‡	121.4 ± 7.5	117.6 ± 5.1	116.1 ± 4.7	0.502

LDL: low-density lipoprotein. †LDL cholesterol levels are given as least-square means ± S.E. adjusted for age and body mass index; ‡LDL cholesterol levels are given as least-square means ± S.E. adjusted for age, body mass index, habitual smoking, coffee consumption and antihypertensive medication use. Bonferroni correction for multiple comparisons was applied. *P < 0.05 vs. Non- or ex-drinkers with Mt5178C; **P < 0.01 vs. Non- or ex-drinkers with Mt5178C.

## Discussion

The present study confirmed a novel gene-environment interaction. Longevity-associated Mt5178 C/A polymorphism and alcohol consumption may combine to modify the risk of hyper-LDL cholesterolemia in middle-aged Japanese men. For men with Mt5178C, habitual alcohol consumption may reduce serum LDL cholesterol levels or risk of hyper-LDL cholesterolemia. However, for those with Mt5178A, alcohol consumption does not appear to influence the risk of hyper-LDL cholesterolemia.

Although our previous analysis was carried out from the viewpoint of gerontology [11], this analysis of gene-environment interactions was undertaken to evaluate the risk of dyslipidemia, which is clinically and preventively important. Therefore, the effect of alcohol on reducing the risk of hyper-LDL cholesterolemia in Mt5178C genotypic men may be more clinically and preventively informative. A previous study reported a significantly negative correlation between alcohol intake and serum LDL cholesterol levels among 193 healthy subjects with Mt5178C [11]. In the present study, among the subjects including patients using antihypertensive medication, alcohol consumption was also significantly and negatively associated with serum LDL cholesterol levels in 239 Mt5178C genotypic men. On multiple logistic regression analysis or analysis of covariance, use of antihypertensive medication was adjusted for.

Individuals with Mt5178C are reported to be more susceptible to atherosclerotic diseases than those with Mt5178A [6-8]. Our results suggest that, for Mt5178C genotypic men, alcohol intake decreases serum LDL cholesterol levels and reduces the risk of hyper-LDL cholesterolemia, which is a crucial risk factor for coronary heart disease [13]. However, for men with Mt5178C, daily alcohol consumption increases the risk of hypertension [4] and hyperuricemia [12]. From the viewpoint of preventing atherosclerotic diseases, it is difficult to determine whether alcohol intake is beneficial for Mt5178C genotypic men. In contrast, individuals with Mt5178A are genetically more resistant to atherosclerotic diseases than those with Mt5178C. For men with Mt5178A, alcohol intake decreases serum triglyceride levels [11]. However, daily alcohol consumption is positively associated with yearly changes in serum LDL cholesterol levels in men with Mt5178A [16]. It is also unclear whether alcohol consumption is favorable for Mt5178A genotypic men.

Combined effects of Mt5178 C/A polymorphism and coffee consumption on risk factors for atherosclerotic diseases have been reported previously [17,18]. For men with Mt5178C, coffee consumption appears to reduce the risk of hypertension [17] and abnormal glucose tolerance [18], and may thus reduce the risk of atherosclerotic diseases. Whereas coffee intake appears to provide antiatherogenic benefits for Mt5178C genotypic men, for Mt5178A genotypic men, coffee consumption appears to increase the risk of hyper-LDL cholesterolemia [19]. Using genotyping of Mt5178 C/A, integration of information regarding alcohol or coffee consumption may contribute to personalized prevention of life-threatening cardiovascular or cerebrovascular diseases.

Although the association between increasing alcohol intake and decreasing serum LDL cholesterol levels is not apparently modified by alcohol dehydrogenase genotype [20], Corella *et al. *reported that, among men with apolipoprotein E gene (APOE) *ε2*, serum LDL cholesterol levels were significantly lower in drinkers than in non-drinkers [21]. In addition to APOE *ε2/ε3/ε4 *allele types, several polymorphisms, such as apolipoprotein A5 gene polymorphism [22], apolipoprotein C-III gene polymorphism [23], peroxisome proliferator-activated receptor α gene polymorphism [24], cholesteryl ester transfer protein *Taq*1B polymorphism [25] and promoter polymorphism of hepatic lipase gene [26], influence the effects of alcohol consumption on serum LDL cholesterol concentrations. Therefore, gene-gene-environment or gene-gene-gene-environment interactions with serum LDL cholesterol levels should be investigated. Further genetic epidemiological investigations will establish individualized alcohol drinking habits in terms of optimizing serum LDL cholesterol concentrations.

The mechanisms underlying the joint effects of Mt5178 C/A (ND2-237 Leu/Met) polymorphism and alcohol consumption on the risk of hyper-LDL cholesterolemia remain unknown. They presumably depend on the amino-acid-related biochemical differences in NADH dehydrogenase in response to alcohol between ND2-237Leu and ND2-237Met. In any case, clarification of the mechanisms of the combined effects of Mt5178 C/A (ND2-237 Leu/Met) polymorphism and alcohol consumption on serum LDL cholesterol concentrations remains a matter for further molecular biological investigation.

Chi-squared test did not reveal a significant difference in the frequency of Mt5178A between this study and other molecular epidemiological studies [27], thus suggesting that there is no genetic bias in the subjects in this study. However, there may have been selection bias due to the recruiting of subjects from among those visiting the hospital for regular medical check-ups. Moreover, the sample size was not sufficient to test the research hypothesis. Although cross-sectional studies can provide a suggestive causality, they cannot establish solid causal links. Therefore, in order to resolve these problems, a large-scale population-based follow-up study is necessary.

Another limitation of this study was the evaluation of habitual alcohol intake based on the frequency of alcohol consumption. As there is a J-shaped curve for the relationship between volume of alcohol consumption and risk of coronary heart disease [28] or ischemic stroke [29], evaluation of volume of alcohol consumption is of great import. Although we have also used this evaluation in previous studies [4,11,12,16,30], whether there is any joint effect between Mt5178 C/A polymorphism and volume of alcohol intake on the risk of hyper-LDL cholesterolemia warrants further investigation. Männistö *et al. *reported that the percentage of energy from fat was significantly lower in male abstainers than in male alcohol drinkers [31]. Therefore, dietary information is required in order to precisely evaluate the relationship between alcohol consumption and serum LDL cholesterol concentration. In this study, LDL cholesterol levels were calculated by means of Friedewald's formula [15]. Therefore, direct measurement of serum LDL cholesterol levels is desirable.

In conclusion, a joint effect of longevity-associated Mt5178 C/A polymorphism and habitual alcohol consumption on the risk of hyper-LDL cholesterolemia was observed in middle-aged Japanese men. For men with Mt5178C, alcohol intake may reduce the risk of hyper-LDL cholesterolemia. Considering that daily alcohol consumption increases other risk factors of atherosclerotic diseases for Mt5178C genotypic men [4,12], it is difficult to determine whether habitual drinking is preventively beneficial. Although further investigation will be required to establish the individualized optimum for maximizing the risk reduction and minimizing the risk increase of habitual alcohol drinking for atherosclerotic diseases, this information regarding the Mt5178 C/A polymorphism will probably contribute to personalized prevention for cardiovascular or cerebrovascular events.

## Competing interests

The authors declare that they have no competing interests.

## Authors' contributions

TK analyzed the data and drafted the manuscript; AK designed the study, carried out the epidemiological survey, carried out the genotyping, analyzed the data, and drafted the manuscript; MI collected the samples; NM helped to carry out the genotyping; KK and MY carried out the epidemiological survey; NS, TO, TS, HO, TI, and HH assisted in data analysis and helped with interpreting the results; YT designed the study and carried out the epidemiological survey. All authors have read and approved the final manuscript.
